# Inactivation of Foodborne Viruses by High-Pressure Processing (HPP)

**DOI:** 10.3390/foods10020215

**Published:** 2021-01-21

**Authors:** Alexander Govaris, Andreana Pexara

**Affiliations:** Laboratory of Hygiene of Foods of Animal Origin, Faculty of Veterinary Science, University of Thessaly, 224 Trikalon Street, 43100 Karditsa, Greece; agovaris@vet.uth.gr

**Keywords:** foodborne viruses, high-pressure processing (HPP), inactivation, non-thermal food-processing technologies

## Abstract

High-pressure processing (HPP) is an innovative non-thermal food preservation method. HPP can inactivate microorganisms, including viruses, with minimal influence on the physicochemical and sensory properties of foods. The most significant foodborne viruses are human norovirus (HuNoV), hepatitis A virus (HAV), human rotavirus (HRV), hepatitis E virus (HEV), human astrovirus (HAstV), human adenovirus (HuAdV), Aichi virus (AiV), sapovirus (SaV), and enterovirus (EV), which have also been implicated in foodborne outbreaks in various countries. The HPP inactivation of foodborne viruses in foods depends on high-pressure processing parameters (pressure, temperature, and duration time) or non-processing parameters such as virus type, food matrix, water activity (a_w_), and the pH of foods. HPP was found to be effective for the inactivation of foodborne viruses such as HuNoV, HAV, HAstV, and HuAdV in foods. HPP treatments have been found to be effective at eliminating foodborne viruses in high-risk foods such as shellfish and vegetables. The present work reviews the published data on the effect of HPP processing on foodborne viruses in laboratory media and foods.

## 1. Introduction

In recent years, viral foodborne diseases have increased worldwide [1]. In the European Union (EU), for the first time, the most common etiological agent of foodborne outbreaks was the viruses in 2014 [2].

Several viruses have been implicated in foodborne outbreaks in various countries, while human norovirus (HuNoV) and hepatitis A virus (HAV) have been recognized as highly infectious and involved in a high number of recorded foodborne outbreaks. HuNoV has been characterized as the leading agent of acute gastroenteritis in many countries, while HAV has been recorded as the leading cause of viral hepatitis, with 1.4 million annual cases worldwide [3]. Human rotavirus (HRV) has been associated with several cases of childhood gastroenteritis [4]. The consumption of shellfish, vegetables, and fruits was linked to the majority of the foodborne viral outbreaks. Hepatitis E virus (HEV) infections were initially associated with drinking contaminated water, but several cases were also attributed to contaminated food such as undercooked meat and meat products in certain outbreaks globally [5]. Other enteric viruses such as human astrovirus (HAstV), human adenovirus (HuAdV), Aichi virus (AiV), sapovirus (SaV), and enterovirus (EV) are also notable foodborne viruses, causing sporadic outbreaks worldwide [6]. The description and classification of foodborne viruses are summarized in Table 1.

Foodborne viruses can survive for a long time in foods without loss of infectivity [6]. For example, they can survive for several months in shellfish [7]. Certain traditional preservation methods such as smoking or drying may not be effective for foodborne viruses’ inactivation in foods [1].

Among traditional preservation methods, thermal processing methods such as sterilization are effective techniques since they are able to inactivate highly resistant spore forming bacteria as well as viruses [8,9]. Despite the food safety benefits, thermal processing methods can also affect the quality characteristics of foods by degrading their nutritional and sensory properties [10].

In recent years, consumers prefer healthier foods of high quality [11]. Several foodborne viruses’ outbreaks have also been associated with the consumption of minimally processed and ready-to-eat foods. Thus, alternative preservation methods should be applied for the inactivation of viruses in food [6]. Novel nοn-thermal food-processing technologies, including high-pressure processing (HPP), cold plasma (CP), ultraviolet light (UV), irradiation, and pulsed electric field (PEF), have been examined for foodborne viruses’ inactivation, parallel to the maintenance of the sensory and nutritional characteristics of the treated food products [6]. One of the most promising novel non-thermal food-processing methods is HPP. The HPP method applies instantaneous hydrostatic pressure uniformly to the food products. The initial studies of HPP in foods were focused on inactivating foodborne vegetative bacteria, fungi, and protozoa [12]. However, in recent years, HPP technology has also been examined concerning the elimination of viruses in foods [13,14,15,16,17,18].

The objective of this work was to review the HPP’s effect on foodborne viruses’ inactivation in laboratory media and foods in the last decade.

## 2. High-Pressure Processing (HPP)

The effect of high pressure on the inactivation of microorganisms in foods has been examined for several years. In 1899, the inactivation of microorganisms in milk by applying high pressure (600 MPa) was initially reported by Hite in the USA [19]. Due to a lack of appropriate high-pressure equipment and packaging materials, the HPP method was not commercialized for almost a century. Research on appropriate HPP equipment for food applications started in 1980. In 1992, the HPP method in foods was successfully commercialized in Japan and over the past few years, HPP-treated foods have been commercialized in several countries [20].

HPP applies isostatic pressure (300–600 MPa) to liquid or solid foods, packaged or unpackaged, at temperatures varying from below 0 to above 100 °C, but usually at chilled or mild process temperatures (<45 °C), for a few seconds to over 20 min [6,21]. In HPP treatment, foods are packaged in flexible polymer-based materials, such as films and vacuum pouches, and semi-rigid packaging such as flasks and bottles, and are placed in a pressure vessel usually filled with water, which is used for the pressure transmission to the food [22]. Τhe volume of foods decreases under pressure and increases during decompression. In HPP treatment of packaged foods, post process contamination is avoided because food products are found in their final package [21]. HPP methods are also used for bulk liquids before packaging (in bulk) and food products are packaged aseptically after processing to ensure shelf stability [22].

High-pressure application to foods can cause physical and chemical changes. The HPP effect on foods is governed by the Le Chatelier, isostatic pressing, and microscopic ordering principles. The Le Chatelier principle states that any chemical reaction, phase transition, and molecular configuration change associated with a decrease in volume is enhanced, while the volume is not increased. This means that covalent bonds are not broken by HPP and low weight molecules such as vitamins or the aroma compounds of foods are usually not affected [23]. In contrast, macromolecules, such as carbohydrates and proteins, can have their native structure modified by HPP application, and this may lead to additional functional properties for foods [24]. According to the principle of microscopic ordering, at a stable temperature, an increase in pressure results in an increase in the degrees of ordering of molecules of a certain substance. The temperature and pressure show antagonistic forces on chemical reactions and molecular structure. Thus, the temperature is increased as the pressure is increased (ca 2–3 °C per 100 MPa in foods) [25]. The isostatic pressing principle (Pascal’s law) states that the pressure applied to any part of an enclosed material will be transmitted uniformly in all directions of the material.

The majority of HPP food products are processed using batch process systems. A typical HPP batch system consists of four basic components: the pressure vessel, pressurization fluid, pressure intensifier, and pump [20]. No additional energy is required as soon as the desired pressure is achieved in a HPP process [26]. HPP is also a rather environmentally friendly food process technology [27].

The HPP method is able to inactivate microorganisms either in the food matrix or on the food surface [23]. Nowadays, HPP applications in the food industry includes a variety of foods products such as shellfish, meat and ready-to-eat meat products, vegetable products, juices, jams, and yogurt [26].

HPP regulatory safety criteria for foods and for the labeling of HPP-treated foods have been set in many countries worldwide [27]. In the EU, HPP-treated food products should be appropriately labeled and comply with the requirements of Regulation (EC) No. 2017/2470 and Regulation (EU) 2015/2283 for commercialized novel foods [28]. In the USA, the HPP process was approved by Department of Agriculture (USDA) and the Food and Drug Administration (FDA) and similar process requirements were set for traditional thermal pasteurization [29]. The US National Advisory Committee on Microbiological Criteria for Foods (NACMCF) listed HPP as a supplementary non-thermal pasteurization technique [27,30].

### HPP Effect on Food Quality

HPP can affect the quality properties of foods by increasing or decreasing quality attributes. HPP’s positive or negative effects on the quality characteristics of foods depend on the food type and the HPP treatment [31].

Since HPP does not affect covalent bonds, it presents a minimal change to food components [29,32]. As mentioned earlier, important low molecular weight components of foods, such as vitamins, are almost kept stable during HPP treatment [33,34]. However, non-covalent bonds such as hydrogen, hydrophobic bonds, and ionic bonds are changed by HPP. Thus, HPP may change macromolecules such as the carbohydrates and proteins of foods and change their physicochemical and functional properties. High pressure can affect the tertiary and quaternary structures of proteins and cause irreversible structural protein changes in foods, with gelation, denaturation, or aggregation modifications of the proteins. Thus, HPP can destroy enzymes and certain allergenic proteins in foods. HPP can also modify the chemical bonds of carbohydrates and cause their gelatinization, which is similar to the thermal gelation of carbohydrates and starch swelling in foods [24].

HPP is commercially used for the sterilization of human milk since it provides better conservation of sensitive nutrient components (e.g., vitamins C and E) as compared to thermal sterilization, as well as inactivation of viruses and spore forming bacteria (e.g., *Bacillus cereus*) [35].

HPP can result in the discoloration of raw meat due to changes from myoglobin oxidation. Myoglobin is prone to oxidation due to heme, a ferrous component, which is released during the HPP treatment and can be easily oxidized in an oxygen atmosphere. The color of HPP-treated raw red meat, which is rich in myoglobin (e.g., beef or lamb), is changed drastically to a color similar to that of cooked meat. However, the color of white meat (e.g., poultry) or cured meat is less affected by HPP treatment [21,36]. In addition, the myofibrillar proteins of raw red meat may be also aggregated by a HPP treatment and result in a change in the meat’s surface reflectance and increase the meat’s lightness. However, the HPP treatment may be beneficial for tenderization and conditioning of the meat due to the protein’s changes in the meat [37]. HPP may also cause the crystallization of lipid components and consequently, enhance lipid oxidation, particularly in rich lipid foods [38]. HPP treatment may also promote lipid oxidation in raw meat due to increased free radical formation at pressures higher than 500 MPa [36].

HPP can change plant matrix structures and lead to the disruption of plant cell walls, resulting in the release of phenolic compounds with antioxidant properties into the extracellular environment [39,40]. Thus, the antioxidant activity of HPP-treated blackberry and strawberry purées was higher than those of the thermally processed one [41]. In plant foods, the release of antioxidant and anti-inflammatory components such as carotenoids and glucosinolates into the extracellular environment may be increased by the HPP treatment as their extractability is enhanced. The bioavailability and extractability of nutrients caused by the matrix disruption of foods depend on the HPP treatment process [32,42]. The effects of HPP on food components could lead to the development of new products. For example, the increased formation of actomyosin in HPP-treated meat, especially at a low salt concentration, can result in the production of new types of meat products [37].

## 3. Foodborne Viruses’ Inactivation by HPP

The majority of studies on the effect of HPP treatment on microorganisms in foods were conducted for bacteria, and fewer studies were established for foodborne viruses [12,43]. However, studies on the effect of HPP treatment on foodborne viruses in foods have increased in recent years [13,14,15,16,17,18]. The initial study of HPP treatment on viruses’ sensitivity was conducted in 1929, by examining a non-enveloped plant virus, the tobacco mosaic virus (TMV) [44], while Basset et al. [45] reported that a HPP treatment of 800 MPa for 45 min proved adequate for the inactivation of TMV.

### 3.1. Factors Affecting Foodborne Viruses’ Inactivation by HPP

The HPP treatment can disrupt the capsid proteins of the viruses and disable the attachment and entrance of the virions into infected host cells. In enveloped viruses, HPP can also denature or destroy the envelop and cause a loss of virus infectivity [46]. The effectiveness of HPP to inactivate viruses as well as foodborne viruses in foods depends on factors related to high-pressure processing parameters (particularly pressure, temperature, and duration time) or non-processing parameters (virus type, food matrix, water activity (a_w_), and pH of foods).

#### 3.1.1. Processing Parameters

An increase in pressure or temperature of the HPP process can enhance the inactivation of viruses. Several combinations of HPP pressure and time levels on a virus’ inactivation effects have been examined [26]. HAV was decreased by 1 and 2 log after a HPP treatment of 300 and 400 MPa for 600 s, respectively, while it was decreased below the detection limit within a HPP treatment of 500 MPa for 300 s [47]. It has been indicated that an increase in HPP pressure level can result in a higher inactivation of viruses as compared to those of an increase in HPP time [48,49]. Kingsley et al. [50] reported a gradual inactivation of Murine norovirus 1 (MNV-1), a surrogate for HuNoV, under a HPP pressure of 350 to 450 MPa at 20 °C for 5 min, while inactivation of the virus was achieved at the highest 450 MPa HPP treatment. Sanchez [51] reported that HPP treatments higher than 400 MPa are effective for the inactivation of HAV. Chen et al. [48] found that a HPP treatment of 200 MPa at ambient temperature for 20 min caused a 2.8 log reduction in Feline calicivirus (FCV), another surrogate for HuNoV, while an elongation of the holding time to 72 min caused only an additional 0.9 log reduction in the same virus. Lou et al. [52] reported that MNV and FCV, surrogates for HuNoV, were inactivated at HPP treatments of 500 to 600 MPa for 2 min, but these HPP treatments were not efficient at destroying the capsid of HuNoV, even at a 60 min holding time HPP. Other studies concluded that a longer time of the HPP treatment can also increase the inactivation of viruses. FCV was decreased by 3.6 log after a HPP treatment of 300 MPa for 120 s and was undetectable after a HPP treatment of 300 MPa for 180 s [47]. However, HuNoV was degraded at a higher rate by increasing HPP pressure levels than increasing HPP time levels [52].

The capsid of HuNoV is quite resistant at high pressure HPP treatments [52,53]. In laboratory growth media, HAV and HRV viruses were inactivated by HPP treatments at 450 MPa at 22 °C for 5 min and 300 MPa at 25 °C for 2 min [39,54]. AiV is also resistant to HPP treatment. AiV was found fully infectious in a laboratory growth medium after a HPP treatment at 600 MPa for 5 min [55].

HPP temperature can act either synergistically or antagonistically with pressure for the inactivation of specific target viruses. HuNoV and its surrogates MNV and Tulane virus (TV) were easily inactivated by HPP treatment at a refrigerated temperature (4 °C) than at ambient temperatures [50,56,57,58]. In contrast, inactivation of HAV by HPP treatment was achieved at ambient or higher temperature [43,59,60]. Both HuNoV GI.1 and GII.4 strains treated by high hydrostatic pressure (HPP) at temperatures ranging from 1 to 35 °C showed increasing pressure sensitivity by decreasing temperatures of HPP treatment [61]. FCV was not affected by HPP pressure at ambient temperatures; however, FCV was inactivated when HPP pressure was applied at 4 °C or higher than 30 °C [48,50]. HPP treatment at 450 MPa for 5 min at 1 °C enhanced the inactivation of HuNoV in oysters as compared to the same HPP treatment at 25 °C [16].

#### 3.1.2. Non-Processing Parameters

Several studies have indicated that the resistance of foodborne viruses to HPP treatment is associated with the virus type. Poliovirus (PV), used as a surrogate for HAV, showed no inactivation at a HPP treatment of 600 MPa for 1 h, but adenoviruses were inactivated at HPP pressures higher than 400 MPa [62]. Grove et al. [47] reported that PV was quite resistant with low or no substantial decrease in titer after a HPP treatment at 600 MPa for 300 s, in contrast to higher inactivation rates of HAV and FCV at HPP treatments with lower HPP pressure. In salsa, FCV, HAV, and murine norovirus (MNV) were inactivated by HPP treatments at 250, 400, and 500 MPa (9 °C) for 1, 5, and 10 min, respectively [63].

In general, enveloped viruses are less resistant to non-enveloped viruses during a HPP treatment [52]. The lipid and protein components of the envelope are less stable at HPP treatment than nucleic acids, particularly at refrigerated temperatures [43]. The protein denaturation of the envelope is due to the destabilization of the hydrogen bonds and the changes in the hydrophobic groups, causing a loss of tertiary and quaternary structure of the proteins [64]. HPP treatment of 300 MPa for 10 min at 25 °C eliminated the enveloped viruses of herpes simplex virus type 1 (HSV-1) and human cytomegalovirus (HCMV) in laboratory media [65], in contrast to survival of the non-enveloped PV at a HPP treatment of 600 MPa for 60 min at 25 °C [66].

Various strains of the same virus may show different inactivation sensitivities to a HPP treatment [57]. HuNoV strains (GI.1 and GII.4) were examined under different pressure HPP treatments and found that the GI.1 strain presented a higher resistance to pressure than the GII.4 strain [56,62]. Araud et al. [57] examined seven strains of four serotypes of HRV and observed that the strains showed a different resistance profile to various pressure and temperature combinations of HPP, while the most resistant strain was found in a strain (WA) derived from oysters, which was eliminated at a pressure treatment of 400 MPa for 2 min. Four HuNoV strains exhibited different sensitivities to HPP pressures ranging from 200 to 600 MPa [56].

Previous studies mentioned the importance of the pH of foods for the stability of the viruses during HPP treatment. HPP treatment can result in a higher decrease in HAV under acidic than neutral conditions [25]. Kingsley and Chen [60] reported that HAV inactivation in the HPP treatment of 400 MPa for 1 min was 2.9 log higher at pH 3 than pH 7 in buffer solutions. In contrast, the MNV-1 virus in a water medium in the HPP treatment (350 MPa for 2 min at 4 °C) was completely inactivated (8-log reductions) at pH 7, whereas only a 6.0 log virus decrease was achieved at pH 4 [49]. GI.1 and GII.4 strains of norovirus were more resistant to HPP treatments at pH 4 than at pH 7 [67]. Similarly, HuNoV, HRV, FCV, and MNV-1 were easily inactivated by HPP treatments at neutral pH than at low pH [48,56,58,68,69]. The mechanism of the pH effect on the HPP pressure inactivation of various non-enveloped viruses is unknown and may be dependent on the capsid protein structure of the viruses [26].

The inactivation rate of viruses in HPP treatment is influenced by the a_w_ and the NaCl content of the foods [13]. The viruses show a better stability in water than in a dry environment [1]. Survival of viruses is generally higher in foods with high a_w_ than in the low moisture foods [70]. In an equivalent a_w_ of culture media with sucrose (0–40%) and NaCl (0–12%) supplementing content, FCV was decreased during HPP treatment to a larger extent in the sucrose than the NaCl solution [68]. The presence of salt in foods may play a protective role against the inactivation of viruses by a HPP treatment [13]. In a laboratory medium supplemented with 0 or 12% NaCl and processed by HPP at 250 MPa for 5 min, FCV was significantly decreased by 5 and 0.7 log, respectively. In a buffer supplemented with 0, 1, 3, or 6% NaCl and treated by HPP of 400 MPa at 50 °C for 1 min, the HAV was decreased by 4, 4.1, 1.3, and 0.4 log, respectively [60]. This protective role of NaCl may be due to interactions of the NaCl with the virus proteins resulting in a change of the natural hydrophilic form of the proteins to a hydrophobic form of them under HPP pressure, change of the water salvation cage of the viral proteins, loss of water content, and change of the compression ability of the foods [63]. The NaCl may also stabilize the capsid proteins of viruses in the HPP treatment, a fact that should be considered during the HPP treatments of shellfish products [66].

The HPP effect on viruses is also dependent on the food type. Food components such as lipids, carbohydrates, or proteins may play a protecting role in the inactivation behavior of viruses [71]. The same virus may be inactivated by a HPP treatment in a different manner in various food products [46,72]. FCV and bovine enterovirus (BEN), a surrogate for HAV, showed a higher resistance during HPP treatments in mussels and oysters, as compared to seawater and culture medium [73]. The same HPP treatments (400 MPa for 10 min) resulted in a higher reduction in FCV and MNV viruses in swine liver than ham [46]. The HPP treatments of 350 and 400 MPa for 5 min resulted in HAV decreases of 2.1 and 3.6 log in blue mussels, respectively, and 1.7 and 2.9 log in Mediterranean mussels, respectively [74]. At 250 MPa for 2 s treatment, FCV-F9 was decreased by 1.77 and 0.78 log PFU/mL in orange juice and milk, respectively [75]. At 300 MPa for 2 s treatment, MNV-1 showed decreases of 1.32 and 2.5 log PFU/mL, respectively [75]. The infectious bursal disease virus (IBDV) during a HPP treatment of 600 MPa for 10–15 s at ambient temperature showed decreases of 5–6 log and 0.5–2 log in chicken meat homogenate and eggs, respectively [76]. Nasheri et al. [15] reported that at HPP of 600 MPa for 5 min, HEV was decreased by 2 and 0.5 log in laboratory media and pork pâté, respectively, and concluded that HPP treatment may not be sufficient to mitigate the risk of HEV in pork pâté.

MNV-1 showed higher decreases in laboratory medium (8 log) than in strawberries (5.8 log) and strawberry puree (4.7 log) during the HPP treatment of 450 MPa for 2 min at 4 °C [69]. It is also important to note that HPP applied to plant cells facilitates the release of phenolic components, which exhibit antiviral activities. Thus, a HPP treatment of vegetables or fruits may enhance virus inactivation due to a higher phenolic activity [58]. 

The published data on the effectiveness of HPP treatment on the inactivation of foodborne viruses/surrogates in laboratory media and foods are summarized in Table 2 and Table 3, re-spectively.

## 4. Conclusions

In conclusion, HPP was found to be an effective non-thermal food processing technology for the inactivation of foodborne viruses such as HuNoV, HAV, HAstV, and HuAdV in foods under certain parameters and conditions and depending on the type of food. An increase in pressure or temperature of HPP can enhance the inactivation of viruses. The resistance of the foodborne viruses to HPP treatment is associated with the virus type. The survival of viruses is generally higher in foods with high a_w_ than in the low moisture foods during HPP treatment. The effect of the pH of foods on the resistance of HPP treatment depends also on the virus type. The presence of salt in foods may play a protective role against the inactivation of viruses by a HPP treatment. The food components may also protect viruses during HPP treatment as compared to laboratory media. HPP treatment was found to be effective in eliminating foodborne viruses in high-risk foods such as shellfish and vegetables. However, further studies are required to assess the proper HPP conditions for the inactivation of foodborne viruses in foods. The increased knowledge of HPP treatment of foods and the development of appropriate high-pressure equipment and packaging materials will enhance the commercial application of HPP in foods in future.

## Figures and Tables

**Table 1:** Foodborne viruses.

Viruses	Particle/Genome ^1^	Genus/Family
Human norovirus (HuNoV)	Non-enveloped/ssRNA	*Norovirus/Caliciviridae*
Human rotavirus (HRV)	Non-enveloped/segmented dsRNA	*Rotavirus/Reoviridae*
Hepatitis A (HAV)	Non-enveloped/ssRNA	*Hepatovirus/Picornaviridae*
Human astrovirus (HAtVs)	Non-enveloped/ssRNA	*Mamastrovirus/Astroviridae*
Aichi virus (AiV)	Non-enveloped/ssRNA	*Kobuvirus/Picornaviridae*
Hepatitis E (HEV)	Non-enveloped/ssRNA	*Orthohepevirus/Hepeviridae*
Human adenovirus (HAdV)	Non-enveloped/dsDNA	*Mastadenovirus/Adenoviridae*
Sapovirus (SaV)	Non-enveloped/ssRNA	*Sapovirus/Caliciviridae*
Enterovirus (EV)	Non-enveloped/ssRNA	*Enterovirus/Picornaviridae*

^1^ ssRNA—single-stranded RNA; dsRNA—double-stranded RNA; dsDNA—double-stranded DNA.

**Table 2:** Inactivation of foodborne viruses/surrogates during HPP treatments in laboratory media.

Virus/Surrogate Tested ^1^	Treatment Parameters	Medium ^2^	Reduction ^3^	References
HuNoV GII.1-509	200 MPa, 4 °C, 5 min	Distilled H_2_O	1.6 log RNA copies/mL	[56]
	200 MPa, 4 °C, 5 min	PBS	>1.2 1.2 log RNA copy/mL	
	200 MPa, 4 °C, 5 min/pH 7.0	MEM	1.2 log RNA copy/mL	
	200 MPa, 4 °C, 5 min/pH 4.0	MEM	No reduction	
	200 MPa, 4 °C, 5 min/pH 10.0	MEM	1.0 log RNA copy/mL	
	200 MPa, 20 °C, 5 min/pH 7.0	MEM	No reduction	
	200 MPa, 40 °C, 5 min/pH 7.0	MEM	No reduction	
	400 MPa, 4 °C, 2 min/pH 4.0	PBS	2.14 log RNA copy/mL	[58]
	400 MPa, 20 °C, 2 min/pH 4.0	PBS	1.08 log RNA copy/mL	
	400 MPa, 4 °C, 2 min/pH 7.0	PBS	3.06 log RNA copy/mL	
	400 MPa, 20 °C, 2 min/pH 7.0	PBS	1.24 log RNA copy/mL	
HuNoV GII.4-5M	200 MPa, 4 °C, 5 min	Distilled H_2_O	>4-log RNA copy/mL	[56]
	200 MPa, 4 °C, 5 min	PBS	2.7 RNA copy/mL	
	200 MPa, 4 °C, 5 min/pH 7.0	MEM	2.43 log RNA copy/mL	
	200 MPa, 4 °C, 5 min/pH 4.0	MEM	0.58 log RNA copy/mL	
	200 MPa, 4 °C, 5 min/pH 10.0	MEM	1.77 log RNA copy/mL	
	200 MPa, 20 °C, 5 min/pH 7.0	MEM	0.1 log RNA copy/mL	
	200 MPa, 40 °C, 5 min/pH 7.0	MEM	0.26 log RNA copy/mL	
	200 MPa, 4 °C, 5 min/pH 7.0	MEM	2.43 log RNA copy/mL	
	200 MPa, 4 °C, 5 min/pH 4.0	MEM	0.58 log RNA copy/mL	
	200 MPa, 4 °C, 5 min/pH 10.0	MEM	1.77 log RNA copy/mL	
	200 MPa, 20 °C, 5 min/pH 7.0	MEM	0.1 log RNA copy/mL	
HuNoV GII.6-490	200 MPa, 4 °C, 5 min	Distilled H_2_O	2 log RNA copies/mL	[56]
	200 MPa, 4 °C, 5 min	PBS	2 log RNA copy/mL	
	200 MPa, 4 °C, 5 min/pH 7.0	MEM	1.3 log RNA copy/mL	
	200 MPa, 4 °C, 5 min/pH 4.0	MEM	No reduction	
	200 MPa, 4 °C, 5 min/pH 10.0	MEM	0.9 log RNA copy/mL	
	200 MPa, 20 °C, 5 min	MEM	No reduction	
	200 MPa, 40 °C, 5 min	MEM	No reduction	
HAV	450 MPa, ambient temp., 5 min	DMEM	7.0 log PFU/mL	[43]
	400 MPa, 50 °C, 1 min	DMEM-FBS	4.0 log PFU/mL	[60]
	400 MPa, 50 °C, 1 min, 1% NaCl	DMEM-FBS	4.1 log PFU/mL	
	400 MPa, 50 °C, 1 min, 3% NaCl	DMEM-FBS	1.3 log PFU/mL	
	400 MPa, 50 °C, 1 min, 6% NaCl	DMEM-FBS	0.4 log PFU/mL	
HRV	300 MPa, 4 °C, 2 min/pH 7.0	MEM	4.1 log PFU/mL	[49]
	300 MPa, 4 °C, 2 min/pH 4.0	MEM	1.9 log PFU/mL	
	400 MPa, 4 °C, 2 min	MEM	5 log PFU/mL (K8 strain) >5 log PFU/mL (Ku strain)	[57]
	450 MPa, 4 °C, 2 min	MEM	>5 log PFU/mL (S2 strain) 5.3 log PFU/mL (YO strain)	
	450 MPa, 20 °C, 2 min	MEM	1 log PFU/mL (K8 strain) 4.1 log PFU/mL (Ku strain)	
HEV	400 MPa, ambient temp. 1 and 5 min 600 MPa, ambient temp., for 1 and 5 min	MEM + 10% FBS	2 log infectious particles	[15]
AiV	600 MPa, ambient temp., 5 min	MEM + 2% FBS MEM + 10% FBS	No reduction	[55]
Poliovirus	600 MPa, 20 ◦C, 60 min	EMEM	No reduction	[62]
Coxsackie virus (CAV9)	400 MPa, ambient temp., 5 min	MEM + 10% FBS	3.4 log TCID50	[55]
	500 MPa, ambient temp., 5 min	MEM + 10% FBS	6.5 log TCID50	
	500 MPa, ambient temp., 5 min	MEM + 10% FBS	7.6 log TCID50	
Coxsackie virus (CBV5)	600 MPa, ambient temp., 5 min	MEM + 10% FBS	No reduction	[55]
FCV	200 MPa, 10 °C, 4 min	DMEM-FBS	5.0 log PFU/mL	[48]
	200 MPa, 20 °C, 4 min	DMEM-FBS	0.3 log PFU/mL	
	250 MPa, 20 °C, 1 min/pH 6.0	DMEM-FBS	4.1 log PFU/mL	[68]
	250 MPa, 20 °C, 5 min, 12% NaCl	DMEM-FBS	0.7 log PFU/mL	
	50–350 MPa, 20 °C, 5 min	DMEM-FBS	3.84 log TCID50 (250 MPa) >5.42 log TCID50 (300 MPa)	[73]
BEN	50–350 MPa, 20 °C, 5 min	MEM	0.33 log TCID50 (250 MPa)	[73]

^1^ HuNoV-Human norovirus; HAV-Hepatitis A virus; HRV-Human rotavirus; HEV-Hepatitis E virus; AiV-Aichi virus; FCV-Feline calicivirus; BEN-Bovine enterovirus. ^2^ PBS—Phosphate-buffered saline; MEM—Minimal essential medium; DMEM—Dulbecco’s modified Eagle medium; FBS—fetal bovine serum; DMEM-FBS—DMEM with 10% FBS; EMEM—Eagle’s Minimum Essential Medium. ^3^ PFU/mL-Plaque-Forming Unit/mL; TCID50; Median Tissue Culture Infectious Dose.

**Table 3:** Inactivation of foodborne viruses/surrogates during HPP treatments in various foods.

Virus/Surrogate Tested ^1^	Treatment Parameters	Food	Reduction ^2^	References
HuNoV GI.1	400 MPa, 6 °C, 5 min	Oysters	1.3 log PFU/mL	[62]
	600 MPa, 1 and 21 °C, 2 min	Dry blueberries	<1 log	[67]
	500 MPa, 1 °C, 2 min	Wet blueberries	2.7 log	
HuNoV GII.4	400 MPa, 6 °C, 5 min	Oysters	3.6 log PFU/mL	[62]
	400 MPa, 4 °C, 2 min/pH 7.0	Strawberry puree	2.29 log RNA copy/mL	[58]
	400 MPa, 20 °C, 2 min/pH 7.0	Strawberry puree	∼1 log RNA copy/mL	
	500 MPa, 4 °C, 2 min/pH 4.0	Strawberry puree	Slight reduction	
	600 MPa, 4 °C, 2 min/pH 4.0	Strawberry puree	No reduction	
HuNoV GII-7	400 MPa, 25 °C, 5 min	Oysters	1.87–1.99 log RNA copy/mL	[77]
HAV	400 MPa, 9 °C, 1 min	Oysters	3.0 log PFU/mL	[17]
	375 MPa, 21 °C, 5 min/pH 3.67	Mashed strawberry	4.3 log PFU/mL	[43]
	375 MPa, 21 °C, 5 min/pH 5.12	Green onions	4.7 log PFU/mL	
	500 MPa, 4 °C, 5 min	Sausage	3.2 log TCID50/mL	[18]
	500 MPa, 20 °C, 1 min/pH 6.07	Oysters	>4.6 log PFU/mL	[60]
	500 MPa, 40 °C, 1 min/pH 6.07	Oysters	>4.6 log PFU/mL	
	500 MPa, 50 °C, 1 min/pH 6.07	Oysters	4.6 log PFU/mL	
	350 MPa, 18–22 °C, 5 min	Mussels	2.1 log PFU/treatment group (blue mussels) 1.7 log PFU/treatment group (Mediterranean mussels)	[74]
	400 MPa, 18–22 °C, 5 min	Mussels	3.6 log PFU/treatment group (blue mussels) 2.9 log PFU (Mediterranean mussels)	
	400 MPa, 5 °C, 5 min	Seafood meat (tuna, cod, shrimp, and clams) Seafood salad (meat with mayonnaise)	Seafood meat: 1.76 log TCID50/g (tuna) 2.92 log TCID50/g (cod) 1.68 log TCID50/g (shrimp) 3.38 log TCID50/g (clams) Seafood salad: 3.30 log TCID50/g (tuna) 2.22 log TCID50/g (cod) 1.44 log TCID50/g (shrimp) 3.01 log TCID50/g (clams)	[72]
	500 MPa, 20 °C, 2 min	Green onions	>4.7 MPN units/plant 2.5 log MPN units/plant (internalized virus) 5.5 MPN units/plant (external virus)	[14]
HEV	400 MPa, ambient temp. 1 and 5 min; 600 MPa, ambient temp., for 1 and 5 min	Pork pâté	0.5 log infectious particles	[15]
HuAdV	500 MPa, 20 °C, 2 min	Green onions	>1.14 log MPN units/plant (internalized virus) >5.8 log MPN units/plant (external virus)	[14]
FCV	200 MPa, 5 °C, 5 min	Seafood meat (tuna, cod, shrimp, and clams) Seafood salad (meat with mayonnaise)	Seafood meat: >4.5 log TCID50/g (tuna) 1.15 log TCID50/g (cod) >4.4 log TCID50/g (shrimp) 3.15 log TCID50/g (clams) Seafood salad: 3.84 log TCID50/g (tuna) 2.22 log TCID50/g (cod) >4.71 log TCID50/g (shrimp) >5.08 log TCID50/g (clams)	[72]
	50–350 MPa, 20 °C, 5 min	Shellfish (mussels, oysters)	1.5 log TCID50 (250 MPa) (oysters, mussel) >3.83 log log TCID50 (300 MPa) (oysters)	[73]
FCV 2280	400 MPa, 10 min	Swine liver	4.2 log	[46]
	600 MPa, 10 min	Ham	4.1 log	
FCV wildtype (wt)	400 MPa, 10 min	Swine liver	≥5.0 log	[46]
	600 MPa, 10 min	Ham	4.4 log	
	500 MPa, 4 °C, 5 min	Sausage	2.89 log TCID50/mL	[18]
BEN	50–350 MPa, 20 °C, 5 min	Shellfish (mussels, oysters)	No reduction (250 MPa) 2.25 log TCID50 (450 MPa) (oysters)	[73]
MS2	500 MPa, 4 °C, 5 min	Sausage	1.46 log TCID50/mL	[18]
	400 MPa, 10 min	Swine liver	No reduction	[46]
	600 MPa, 10 min	Ham	1.7 log PFU/mL	
ΦX174	400 MPa, 10 min	Swine liver	1.1 log PFU/mL	[46]

^1^ HuNoV-Human norovirus; HAV-Hepatitis A virus; HEV-Hepatitis E virus; HuAdV-Human adenovirus; FCV-Feline calicivirus; BEN-Bovine enterovirus; MS2, ΦX174Bacteriophages used as surrogates for HAV and HuNoV, respectively. ^2^ PFU/mL-Plaque-Forming Unit/mL; TCID50; Median Tissue Culture Infectious Dose.

## Data Availability

No new data were created or analyzed in this study. Data sharing is not applicable to this article.

## Abbreviations

AiV	Aichi virus
BEN	Bovine enterovirus
CP	Cold plasma
DMEM	Dulbecco’s modified Eagle medium,
DMEM-FBS	DMEM with 10% FBS
dsDNA	double-stranded DNA
dsRNA	double-stranded RNA
EMEM	Eagle’s Minimum Essential Medium
EV	Enterovirus
FBS	Fetal bovine serum
FCV	Feline calicivirus
FDA	Food and Drug Administration
HAstV	Human astrovirus
HAV	Hepatitis A virus
HCMV	Human cytomegalovirus
HEV	Hepatitis E virus
HPP	High-pressure processing
HRV	Human rotavirus
HSV-1	Herpes simplex virus type 1
HuAdV	Human adenovirus
HuNoV	Human norovirus
IBDV	Infectious bursal disease virus
MEM	Minimal essential medium
MNV-1	Murine norovirus 1
NACMCF	National Advisory Committee on Microbiological Criteria for Foods
PBS	Phosphate-buffered saline
PEF	Pulsed electric field
PV	Poliovirus
SaV	Sapovirus
ssRNA	single-stranded RNA
TMV	Tobacco mosaic virus
UV	Ultraviolet light
